# High Frequency of Deletions in the *pfhrp*2 and *pfhrp*3 Genes of *Plasmodium falciparum* in the Middle Rio Negro Region of the Brazilian Amazon

**DOI:** 10.3390/tropicalmed9070149

**Published:** 2024-07-02

**Authors:** Daniela Romero Bally, Simone da Silva Santos, Diego Calafate Arregue, Mariana Kelly de Mattos, Martha C. Suárez-Mutis

**Affiliations:** 1 Laboratório de Doenças Parasitárias, Instituto Oswaldo Cruz, Fundação Oswaldo Cruz, Rio de Janeiro 21040-900, Brazil; daniela.r.bally@gmail.com (D.R.B.); simone@ioc.fiocruz.br (S.d.S.S.); dicalafate98@gmail.com (D.C.A.); marianakelly29@hotmail.com (M.K.d.M.); 2 Post-Graduation Program in Tropical Medicine, Instituto Oswaldo Cruz-Fiocruz, Rio de Janeiro 21040-900, Brazil

**Keywords:** malaria, *P. falciparum*, Brazilian Amazon, *pfhrp2/3* deletions, rapid diagnostic tests, elimination

## Abstract

Several countries are reporting natural populations of *P. falciparum* with deletions in the *pfhrp*2/3 genes that can lead to false-negative results in rapid diagnostic tests. To investigate the prevalence of deletion in the *pfhrp*2/3 genes in the Rio Negro basin in the Brazilian Amazon and identify whether there is clinical differentiation between individuals infected by these parasites, clinical samples collected from 2003 to 2016 were analyzed from symptomatic and asymptomatic *P. falciparum*-infected individuals. The molecular deletion of *pfhrp2* and *pfhrp3* genes was evaluated using the protocols recommended by the WHO. From 82 samples used, 28 (34.2%) had a single deletion in *pfhrp*2, 19 (23.2%) had a single deletion in *pfhrp*3, 15 (18.3%) had a double deletion (*pfhrp2/3*), and 20 (24.4%) did not have a deletion in either gene. In total, 29.3% of individuals had an asymptomatic plasmodial infection and were 3.64 times more likely to have parasites with a double deletion (*pfhrp*2/3) than patients with clinical malaria (*p* = 0.02). The high prevalence of parasites with *pfhrp*2/3 deletions shows the need to implement a surveillance program in this area. Deletions in parasites may be associated with the clinical pattern of the disease in this area. More studies must be carried out to elucidate these findings.

## 1. Introduction

Despite progress in controlling the disease, malaria is still a global public health challenge, generating a significant impact on the social and economic development of affected countries [1]. The Brazilian Amazon region is an endemic area for malaria, with 139,943 reported autochthonous cases in 2021. Most of these cases (82.9%) were attributed to *Plasmodium vivax*, totaling 116,071 cases. Additionally, there were 21,708 cases (15.5%) reported for *Plasmodium falciparum*, and 2155 cases (1.5%) of mixed malaria caused by both *P. vivax* and *P. falciparum* [2]. Since 2016, the Global Technical Strategy for the Elimination of Malaria 2016–2030 [3] has guided countries’ actions with the vision of a “malaria-free world”. The aim is that malaria will be eliminated in the next two decades. That same year, Brazil also embarked on the initial elimination of *P. falciparum* to end transmission by this parasite by 2030 [4]. Due to the biological characteristics of *P. falciparum*, this would be the most easily eliminated parasite in the medium term, and early diagnosis with appropriate treatment is one of the pillars that can lead to the interruption of transmission [4,5]. The gold standard for diagnosing malaria is the thick smear, requiring a minimum healthcare infrastructure such as good quality microscopes, reagents, and well-trained microscopists [6]. In Brazil, a continental country with more than 4,000,000 km^2^ of Amazon region, many areas are difficult to access and without the minimum healthcare facilities that allows thick smears to be carried out. The development of rapid diagnostic tests (RDTs) in the 1990s allowed the adoption of parasite diagnosis in areas with poor healthcare infrastructure where health workers with minimal training can perform the test and interpret the results [7]. Tests based on identifying *P. falciparum*-specific histidine-rich protein 2 (HRP2 or PfHRP2) are the most used. The histidine-rich protein 3 antigen (HRP3 or PfHRP3), which is also present in *P. falciparum* and shares structural similarities with HRP2, can cross-react with the HRP2 antibody in these tests. The *pfhrp2* gene expresses the HRP2 antigen, while the HRP3 antigen is expressed by the *pfhrp3* gene [8].

In the last decade, false-negative results have been in observed in HRP2-based RDTS in *P. falciparum*-infected individuals due to the deletion of the *pfhrp*2 gene and, consequently, lack of protein expression [9,10,11,12,13,14]. False-negative results delay early diagnosis and treatment. Detecting the presence of *P. falciparum* carrying *pfhrp2/3* deletion and understanding their geographic distribution is justified because early and adequate diagnosis is essential for eliminating the disease in the coming years. Some studies on the prevalence of *pfhrp2* and *pfhrp3* deletion genotypes were done in some localities in Acre, Rondonia, Mato Grosso, Amazonas and Pará states in the Brazilian Amazon basin [14,15]. However, there are large regions that lack these studies and there is no information on the prevalence of these deletions in clinical isolates of *P. falciparum* in the middle of the Negro River, an area of high endemicity for malaria. The objective of this study was to investigate the frequency of deletions in the *pfhrp*2 and *pfhrp*3 genes in *P. falciparum* samples from patients from an area of high epidemiological risk for malaria in the middle Rio Negro, Brazil, and to test for correlations between gene deletions and patient epidemiological profiles and clinical outcomes.

## 2. Materials and Methods

**Figure 1:**
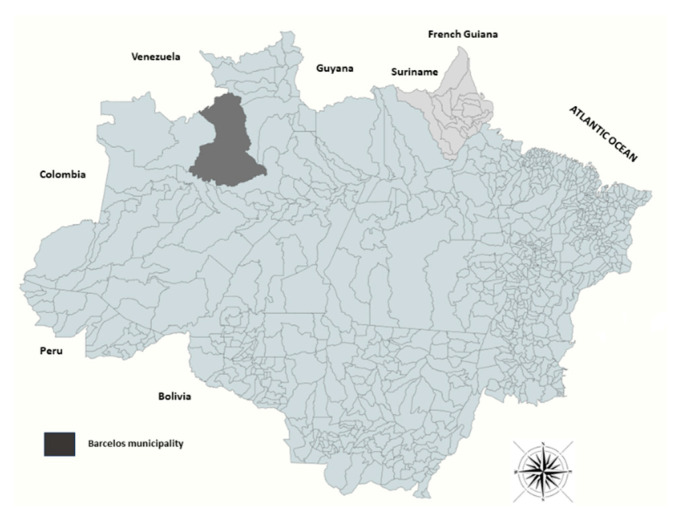
Geographic location of the municipality of Barcelos within the region of the middle Rio Negro, Brazilian Amazon.

A descriptive observational study using samples obtained during several cross-sectional studies conducted in the municipality of Barcelos (AM) between 2003 and 2016 was carried out [16]. The samples were stored in the biorepository of the Parasitic Diseases Laboratory of the Oswaldo Cruz Institute, Fiocruz in Rio de Janeiro. Barcelos belongs to the state of Amazonas and is the second largest municipality in Brazil in terms of size and borders Venezuela (Figure 1). It is considered an area of high epidemiological risk for malaria. With 25,718 inhabitants in 2021, 10,461 malaria cases were reported in the municipality with an Annual Parasitic Index (API) of 40,676 cases by 1000 inhabitants; 1973 of malaria cases were due to *P. falciparum* and 251 due to mixed malaria (*P. falciparum P. vivax*). *P. falciparum* was responsible for 21.26% of cases [2]. Sectional studies carried out in this area have shown a prevalence of asymptomatic plasmodial infection of 20% [16]. The diagnosis of malaria is carried out using thick smears, but RDTs have been increasingly adopted as an alternative method for diagnosing malaria in the municipality. In 2015, only 179 tests were used, while in 2019, 2402 rapid tests were used to diagnose the disease [17].

Sample collection was performed using two strategies: passive and active recruitment. For the former, individuals who presented themselves at health units with symptoms of malaria were invited to participate in this study. Participants who were successfully recruited were also requested to provide their address, and, if granted, an active search for new symptomatic and asymptomatic cases was carried out near that participant’s home. In rural areas, active searches were carried out in all cases. Individuals aged one or more years were eligible. Malaria was defined as any individual coming from Barcelos municipality with a positive thick smear for *P. falciparum* infection and a least one symptom compatible with malaria (fever; chills; sweating; headache). Any person with a positive thick smear or positive PCR for *P. falciparum* without any symptoms of malaria 30 days before or 30 days after sample collection was classified as an asymptomatic carrier [16].

In total, we included 92 samples, buy only 82 had complete data. A capillary blood sample (digital puncture) was collected from all participants and used to perform a thick smear). A microscopist at the Local Malaria Program of the Barcelos Municipal Health Department carried out the parasitological diagnosis. Simultaneously, 5 mL of whole blood in EDTA was collected using a vacutainer tube (Becton, Dickinson^®^, Franklin Lakes, NJ, USA) and transported frozen to the Laboratory of Parasitic Diseases (LDP) at the Oswaldo Cruz Institute/Fiocruz for the molecular experiments.

The DNA was extracted using the Wizard^®^ Genomic Purification Kit (Promega^®^, Madison, WI, USA) from a 300 μL aliquot of whole blood, according to the manufacturer’s protocol [18]. The extracted DNAs was initially used for Polymerase Chain Reaction (PCR) to confirm the diagnosis of *P. falciparum* infection according to the protocol by SNOUNOU et al. [19] with some modifications [16]. The DNA quality from all samples selected for this study was assessed through PCR amplification of the *pfmsp2* gene using the protocol described by SNOUNOU et al. [20] according to recommendations of the World Health Organization (WHO) [13].

Amplifications of exon 2 of the *P. falciparum pfhrp2* and *pfhrp3* genes were performed by semi-nested PCR based on the protocol described by BAKER et al. (2005) [8,21]. A *pfhrp2* and *pfhrp3* deletion case was defined as a sample that was negative in *pfhrp2/3*-specific PCRs (1st and 2nd reactions) but positive by PCR for at least, one of the allelic families of the *pfmsp2* gene. PCR reactions for *pfmsp2* and *pfhrp2/3* were performed in duplicates. DNAs from 3 reference strains of *P. falciparum*: 3D7 (wild type), Dd2 (*pfhrp2* deletion) and HB3 (*pfhrp3* deletion), were used as positive controls in the PCR reactions. To evaluate the sensitivity of the PCR reaction, dilutions of the HB3 and Dd2 control DNA ranging from 0.5 ng/μL to 30 ng/μL were used for *pfhrp2* and *pfhrp3*, respectively. The amplification of diluted samples was possible up to a dilution of 0.5 ng/μL of DNA, and no amplification of the fragment was observed at a dilution of 100 pg/μL of DNA for both genes (results not shown).

Statistical Analysis: The data were stored in an Epi InfoTM 7.2.1.0 program database. Univariate, bivariate analyses and a binomial logistic regression were performed in SPSS 20 (IBM Corp., Armonk, NY, USA). For continuous variables exhibiting a normal distribution, Student’s *t*-test was used to test for statically significant differences between means. For variables that were not normally distributed, a non-parametric test was used (Mann–Whitney test). For categorical variables, the test used was chi-square. Central tendency and dispersion measures were treated as median and interquartile range (IQR) in the case of non-parametric distribution. All statistical analyses used a 5% significance threshold.

## 3. Results

Eighty-two clinical samples from individuals infected with *P. falciparum*, collected between 2003 and 2016, were chosen. PCR amplification of the *pfmsp*2 gene was performed as a criterion for DNA quality control. Among the 82 individuals included, 37 (45.1%) were female, and 45 (54.9%) were male. The median age was 17 years (interquartile range [IQR] = 11.7–34.0), with the minimum age being one year and the maximum being 77 years. The median of previous malaria episodes was five (IQR = 2.75–8). There was no difference between sexes in the median number of prior disease episodes. Only one individual, a female 57 years old, did not report previous malaria episodes. This participant had a clinical malaria. A 46-year-old male reported having had more than 50 previous cases of malaria. This study included 69 individuals with *P. falciparum* infection (84.1%) and 13 with mixed infection (*P. falciparum* + *P. vivax*) (15.8%). This information is found in Table 1.

In total, 58 (70.7%) individuals had clinical signs of malaria at the time of blood collection, and 24 (29.3%) were diagnosed with asymptomatic plasmodial infection by *P. falciparum*. There were no statistical differences in the proportion of individuals with clinical malaria or asymptomatic infection in relation to sex or age. Individuals who reported ten or more previous episodes of malaria were 3.1 times more likely to have asymptomatic infection than those who reported less than ten previous episodes (95% CI: 1.1–9.7); this association was statistically significant (*p* = 0.04).

In individuals with clinical malaria, the number of episodes was negatively associated with age (*p* = 0.03). No statistically significant correlation was found in asymptomatic individuals, probably due to the small number of participants with this condition (24 participants).

In total, 43 samples (52.4%) were found with deletion of the *pfhrp2* gene, of which 28 (34.1%) had a single deletion, and 15 (18.3%) had a double deletion (*pfhrp2/3*). Regarding the *pfhrp3* gene, 34 samples (41.5%) had a deletion, with 19 (23.2%) having a deletion of only the *pfhrp3* gene. Another 20 samples (24.4%) had the wild genotype (they had both genes). The aggregated results are shown in Table 2.

Double deletion *pfhrp*2/3 was found in 12.1% (7/58) of individuals with clinical malaria and 33.3% (8/24) of asymptomatic infected individuals (OR: 3.6, 95% CI: 1.1–11.6; *p* = 0.02). Among the samples without a deletion or with a single deletion, most (51, 76.1%) occurred in participants presenting with clinical malaria, while the remainder (16, 23.9%) occurred in carriers of asymptomatic *P. falciparum* infection.

No differences between individuals with parasites with deletions were found in relation to sex or previous malaria. The median number of previous malaria episodes among individuals infected with double deletion parasites was five (interquartile range = 3.5–12.5). Among individuals infected with parasites without deletion or single deletion, the median of episodes was also five previous malaria cases (interquartile range = 2.0–8.0) (Table 3). Binomial logistic regression showed that the double deletion *pfhrp2/3* was associated only with asymptomatic infection (*p* = 0.02). The other variables did not show statistical significance.

Of three samples collected in 2003, two had a double deletion in *pfhrp2/3* and one was wild type. Of the 58 samples collected in 2006, 23 (39.6%) had an exclusive deletion of *pfhrp2*, 11 (18.9%) had an exclusive deletion of *pfhrp3*, and nine (15.5%) had a double deletion; 15 (38.9%) did not have a deletion of any gene. Of the 18 samples collected in 2007, five had an exclusive deletion of *pfhrp2*, seven had an exclusive deletion of *pfhrp3*, and three had a double deletion; three did not show deletion of any genes. Of the three samples collected in 2016, one was exclusively *pfhrp3* deleted, one had a double deletion, and one had no deletion (Table 4).

## 4. Discussion

The global discussion on eliminating malaria involves the use of tools such as rapid diagnostic tests [1,2,3]. RDTs in Brazil are used when it is difficult to carry out the thick smears, such as in areas of remote access, with poor healthcare structure, and even in urban areas, on night shifts, for example, or in the absence of microscopists (5). Brazil has more than 3000 malaria microscopic diagnostic stations in the Amazon region. However, given the vastness of the endemic areas, in many places, such as indigenous lands, RDTs are the only alternative to conduct an early and adequate diagnosis of febrile individuals [22]. Currently, in addition to thick smears, the Brazilian National Malaria Prevention and Control Program (PNCM) has used SD-BIOLINE MALARIA AG Pf/Pf/Pv^®^ to diagnose malaria, a combined test that detects the HRP2 and pLDH antigens of *P. falciparum* and pLDH from *P. vivax.* It has proven to be quite efficient in supporting malaria diagnosis in the country [22,23].

However, in the last decade, individuals infected with *P. falciparum* with deletion of the *pfhrp2* gene have been more frequently described in different parts of the world [10,24,25]. The deletion of the *pfhrp2* and *pfhrp3* genes in laboratory-adapted *P. falciparum* isolates (clones Dd2 and HB3) was first identified in genetic studies [26,27]. The first evidence of large-scale natural deletion of the *pfhrp*2 and *pfhrp*3 genes was described in 2010 in the Peruvian Amazon [9]. The proportion of parasites without the *pfhrp2* gene increased from 20.7% between 1998 and 2001 to 40.6% in 2003–2005 in the Amazon region of Peru [28]. Isolates with *pfhrp*2/3 deleted were also described in Colombia [12,29], French Guiana [30], Ecuador [31], Bolivia [15], Guyana and Suriname [32], Nicaragua, Guatemala and Honduras [33,34].

Houzé et al. (2011) reported a case of *P. falciparum* malaria in a French citizen who had traveled to the Brazilian Amazon and was misdiagnosed as non-*falciparum* malaria after a negative result for HRP2 in a RDT [35]. It was later proven that this isolate did not possess the *pfhrp2* and *pfhrp3* genes. Another study identified *P. falciparum* isolates with deletions in the *pfhrp*2 and *pfhrp*3 genes through the analysis of 198 isolates from three states in the Brazilian Amazon Basin (Acre, Rondônia, and Pará) collected between 2010 and 2012. It was observed that the state of Acre, bordering Peru, had the highest proportion of *pfhrp*2-negative isolates (31.6%). High proportions of *pfhrp3*-negative isolates were detected in these three Brazilian states, ranging from 18.3% to 50.9% [14].

Our study showed that in the middle Rio Negro region in Brazil, 52.4% of the samples evaluated were *pfhrp2*-negative. This finding is new in the middle Rio Negro basin, in the Amazon region, where there are no published studies on the prevalence of deletion of these genes. When comparing the results of deletions found in Barcelos with studies in other areas of the Amazon Region, for example, in Peru, 41% of samples collected between 2003 and 2007 were negative for *pfhrp2*, where the proportion of mutant parasites in this area has increased over the years [27,35,36]. In the municipality of Cruzeiro do Sul, Acre, 31.6% of the samples collected in 2012 did not have *pfhrp2* amplification, while the other areas evaluated in this study (Bolivia and the states of Pará and Rondônia in Brazil) had a low frequency of parasites with this deletion [14].

Monoclonal antibodies used in HRP2 detection tests often cross-react with the HRP3 antigen. The HRP2 and HRP3 antigens are structural homologs [7]. Cross-reactions, however, are more likely to occur in infections with parasitemias above 1000 parasites per μL of blood, as the HRP3 protein is less expressed than HRP2 [12,37,38]. In our study, the percentage of deletion of the *pfhrp3* gene was 41.5% in the evaluated samples. A deletion frequency of around 70% in Peru was observed [9]. Rachid Viana et al. (2017) found a high prevalence of *pfhrp3*-negative parasites in Acre (38%), Pará (18.3%), Rondônia, and Bolivia (68%) [14]. Few studies in South America and Southeast Asia have observed a high prevalence of parasites with the genes *pfhrp2* deleted and *pfhrp3* present when compared to other genotypes: *pfhrp2*+/*pfhrp3*- and *pfhrp2*-/*pfhrp3*- [33,39]. In the Amazon Region, the prevalence of *pfhrpr3*- parasites is higher than those of *pfhrp2*- [9,14]. Rachid et al. (2017) showed a prevalence of double deletion that fluctuates from 0% in Pará to 26.6% in Acre [14] while the studies by Góes et al. [15] showed a prevalence of more than 90% in the state of Amazonas and in Acre. Little information is available in international border areas and is mainly related to studies carried out in Acre.

It is suggested that, in this region, *pfhrp3*- parasites may have been present for longer than *pfhrp2*- parasites and that *pfhrp3*- parasites have undergone a genetic cross with *pfhrp2*- parasites and produced progeny without both genes [9,27]. In Barcelos, however, a high prevalence of parasites with the *pfhrp2*-/*pfhrp3*+ genotype (34.15%) was found, in addition to *pfhrp2*+/*pfhrp3*- (23.17%) and *pfhrp2*-/*pfhrp3*- (18.29%). In a meta-analysis study carried out by Sepúlveda et al. (2018) [40], it was suggested that the distribution of deletions of the *pfhrp3* gene is associated with areas of transmission of relatively low intensity, even on the African Continent, where the isolated *pfhrp3* deletion is not common. It is possible that *pfhrp3+* parasites may be better adapted to survival within the host. Reduced numbers of deletions of *pfhrp3* are found in African countries where the intensity of transmission of *P. falciparum* is higher than in other places [40].

Although there is much concern about the deletion of the *pfhrp2/3* genes, which can lead to false-negative results from RDT based exclusively on these antigens, the tests used in Brazil detect the HRP2 and pLDH antigens of *P. falciparum* in addition to pLDH of *P. vivax* [8,22]. The use of a test that also detects the *Pf*pLDH antigen can reduce the false-negative rate in the detection of *P. falciparum* isolates that have a deletion of the *pfhrp2/3* genes. Nevertheless, studies have shown that the sensitivity of tests based in *Pf*pLDH is inferior to that of HRP2 [40,41,42]. RDTs based on HRP2, in addition to be more sensitive in general, present superior performance, especially at low parasite densities [43], and have better thermal stability [40]. Therefore, the sensitivity in the isolated detection of the *Pf*pLDH antigen in hot areas with recurrent infections of low parasitemia, such as the municipality of Barcelos and other areas of the Amazon Region, and the presence of parasites with double deletion may reduce the sensitivity of these tests. Furthermore, *Pf*LDH detection RDTs performed better in identifying *P. falciparum pfhrp2*- in regions with high parasite density [44,45].

In a recent recommendation, the WHO indicates the areas in which surveillance of *pfhrp2/3* gene deletions should be prioritized: (1) areas with identification of discordance between RDT based on HRP2 detection and reported microscopy results, (2) areas with unrepresentative or sporadic reports of *pfhrp2/3* deletions in the country, and (3) areas neighboring a locality where frequent *pfhrp2/3* deletions have been identified [44]. This study reports an important scenario of deletions of the *pfhrp2/pfhrp3* genes in the middle Rio Negro region, where the deletion of exon 2 of the *pfhrp2* gene was observed in 52.44% of the samples, in contrast to 24.39% of the samples with amplification of exon 2 of both genes (*pfhrp2*+/*pfhrp*3+). When the samples for this study were collected, the RDTs had not yet been introduced into the PNCM; for this reason, we were unable to perform simultaneous evaluation of RDTs and molecular results. According to the WHO, when the prevalence of *pfhrp2* gene deletions is greater than 5%, HRP2-based RDT should be reviewed as a screening method for diagnosing malaria. If the prevalence is less than 5%, the recommendation is to plan the change over a more extended period [13,46]. However, if the RDT used also has the *Pf*LDH protein, it will be necessary to evaluate the sensitivity of this test in samples with the *pfhrp2/3* deletion.

An interesting finding in our study was the higher prevalence of deletion among asymptomatic individuals: the chance of an asymptomatic individual carrying isolates with a double deletion was higher than among individuals with clinical malaria (3.64 times); this association was not observed in the isolated deletion of one of the two genes studied. The HRP2 and HRP3 antigens are structural homologs [6,7], and the *pfhrp2* and *pfhrp3* genes are probably derived from a common ancestral gene. Therefore, one may compensate for the function of the other [47]. This could explain why there was a difference in the clinic between participants with a double deletion and individuals infected by *P. falciparum* with a single deletion. The association aligns with a study in the Democratic Republic of Congo that sought to explore clinical differences between children infected with parasites with and without deletion of *pfhrp2*. In this study, lower densities of parasites were observed using real-time PCR, a lower proportion of positivity for microscopy, and less fever among individuals infected with *pfhrp2*- plasmodia, although without significant conclusions due to the limited clinical data [48]. The virulence and pathogenicity of parasites deleted for *pfhrp2* and *pfhrp3* genes, and whether these parasites present different patterns of sensitivity to antimalarial drugs than parasites without deletion, needs to be better studied [45,49]. If *pfhrp2* and *pfhrp3* deletions are associated with less virulent infections, there may be a difference in prevalence between symptomatic and asymptomatic infections, and many studies may not be truly representative of the prevalence of deletions in a population or country [12]. Koita et al. (2012) [50] and Houzé et al. (2011) [35] showed the potential of parasites with deletion of the *pfhrp2* and *pfhrp3* genes to cause severe disease. However, it is important to note that the factors that determine whether patients will develop asymptomatic or symptomatic malaria, and/or mild or severe disease, are multiple and not only associated with the genetic characteristics of the parasite. These may include the mosquito inoculation rate, acquired immunity, genetic polymorphisms of the human host, nutritional status of the infected individual, environmental conditions and access to efficient treatment [49]. More studies are needed to clarify these findings.

The actual influence of the genetic diversity of the *pfhrp2* and *pfhrp3* genes on the diagnostic performance with HRP2-based RDTs needs to be better elucidated [51]. Some studies suggest that the genetic variability of *pfhrp2* gene does not seem to be the cause of false-negative results, but rather the high prevalence of deletion of this gene, which is associated with a high rate of false-negative results [38]. Nevertheless, there is evidence suggesting that variations in the amino acid sequence of PfHRP2 could impact the sensitivity of rapid diagnostic tests (RDTs) when parasite densities are extremely low (<200 parasites/mL) [22]. This subject needs to be better clarified.

This study has some limitations, mainly associated with the use of samples that were stored in the laboratory’s biorepository. First, as most samples were collected before the introduction of RDTs in the study area, we were unable to evaluate *pfhrp2/3* deletions with RDT results. Second, at the time of sample collection, the guidelines of the National Malaria Control Program recommended the use of qualitative methods to measure parasitemia and it was not possible to have data on parasitemia. Finally, we did not perform multiclonality studies on these samples.

## 5. Conclusions

Our results highlight the importance of developing genomic surveillance in *P. falciparum* transmission areas, especially in international border areas, together with the evaluation of the effectiveness of RDTs used in the country, as early diagnosis with adequate treatment continues to be one of the pillars that will lead to the elimination of the disease in Brazil.

## Figures and Tables

**Table 1:** Baseline and clinical characteristics of individuals included in this study (*n* = 82).

Variable	Characteristics	*n*	% or [IQR]
Sex	Female	37	45.1%
	Male	45	54.9%
Age (years)	Median [IQR]	17	[11,7–34]
	0–4 years	5	6.2%
	5–15 years	25	30.5%
	≥15 years	52	63.4%
Clinical Phenotype of Malaria Cases	Clinical malaria	58	70.7%
	Asymptomatic infection	24	29.3%
Number of Previous Malaria Episodes	Median [IQR]	5	[2,7–8]
	0	1	1.2%
	1	13	15.9%
	2–4	24	29.3%
	5–9	28	34.1%
	≥10	16	19.5%

[IQR]: interquartile range.

**Table 2:** Results of amplification of exon 2 of the *pfhrp2* and *pfhrp3* genes.

Genotypes	Number of Samples	Percentage (%)
*pfhrp2-/pfhrp3+*	28	34.1
*pfhrp2-/pfhrp3-*	15	18.3
*pfhrp2+/pfhrp3-*	19	23.2
*pfhrp2+/pfhrp3+*	20	24.4
**Total**	**82**	**100**

**Table 3:** Characteristics of individuals infected with parasites with and without deletion of the *pfhrp2* and *pfhrp3* genes.

		Wild Genotype	*pfhrp2* Single Deletion	*pfhrp3* Single Deletion	Double Deletions *pfhrp2/3*	Total
Variables		N = 20 (24.4%)	N = 28 (34.1%)	N = 19 (23.2%)	N = 15 (18.3%)	N = 82 (100%)
		N (%)	N (%)	N (%)	N (%)	N (%)
Gender	Female	7 (18.9%)	16 (43.2%)	8 (21.6%)	6 (16.2%)	37 (45.1%)
	Male	13 (28.9%)	12 (26.7%)	11 (24.4%)	9 (20%)	45 (54.9%)
Age	Median [IQR]	14 [10.5–25.5]	18 [13–34.5]	16 [9–56]	28 [16–38.5]	17 [11.5–34]
	0–4 years	0 (0%)	2 (40%)	2 (40%)	1 (20%)	5 (6.1%)
	5–14 years	11 (40.7%)	8 (29.6%)	7 (25.9%)	1 (3.7%)	27 (32.9%)
	≥15 years	9 (17.3%)	18 (34.6%)	12 (23.1%)	13 (25%)	52 (63.4%)
Clinical Phenotype	Clinical malaria	14 (24.1%)	20 (34.5%)	17 (29.3%)	7 (12.1%)	58 (70.7%)
	Asymptomatic infection	6 (25%)	8 (33.3%)	2 (8.3%)	8 (33.3%)	24 (29.3%)
Previous History of Mild Malaria	Median [IQR]	6.5 [3–10]	4 [1.5–8]	5 [2.5–7]	5 [3.5–12.5]	5 [2.5–8]
	No previous (first malaria)	0 (0%)	1 (100%)	0 (0%)	0 (0%)	1 (1.2%)
	1 episode	2 (15.4%)	6 (46.1%)	3 (23.1%)	2 (15.4%)	13 (15.9%)
	2–4 episodes	5 (20,8%)	8 (33.3%)	6 (25%)	5 (20.8%)	24 (29.3%)
	5–9 episodes	7 (25%)	8 (28.6%)	9 (32.1%)	4 (14.3%)	28 (34.1%)
	≥10 episodes	6 (37.5%)	5 (31.3%)	1 (6.3%)	4 (25.0%)	16 (19.5%)

IQR: interquartile range.

**Table 4:** Genotypes found each year.

Genotype	2003	2006	2007	2016	Total
	n (%)	n (%)	n (%)	n (%)	n (%)
wild	1 (5.3%)	15 (78.9%)	3 (15.8%)	0 (0%)	19 (100%)
*pfhrp2-*	0 (0%)	23 (82.1%)	5 (17.9%)	0 (0%)	28 (100%)
*pfhrp3-*	0 (0%)	11 (57.9%)	7 (36.8%)	1 (5.3%)	19 (100%)
*pfhrp2/3-*	2 (12.5%)	9 (56.3%)	3 (18.8%)	2 (12.5%)	16 (100%)
*Total*	3 (3.7%)	58 (70.7%)	18 (22.0%)	3 (3.7%)	82 (100%)

## Data Availability

De-identified individual participant data presented in this study are available on https://www.arca.fiocruz.br/handle/icict/49203.
